# A kabuli chickpea ideotype

**DOI:** 10.1038/s41598-022-05559-3

**Published:** 2022-01-31

**Authors:** Tuba Eker, Duygu Sari, Hatice Sari, Hilal Sule Tosun, Cengiz Toker

**Affiliations:** 1 Department of Field Crops, Faculty of Agriculture, Akdeniz University, 07070 Antalya, Turkey; 2 Department of Plant Protection, Faculty of Agriculture, Akdeniz University, 07070 Antalya, Turkey

**Keywords:** Plant sciences, Plant breeding

## Abstract

The concept of ‘crop ideotype’ is coined as a desirable plant model expected to better perform for seed yield, oils and other useful characteristics when developed as a cultivar, and it consists of two major approaches, namely, (i) ‘defect elimination’, that is, integration of disease resistance to a susceptible genotype from a resistant genotype and (ii) ‘selection for yield’ by improving yield after crosses between desirable parents. For consideration of these approaches, here we introduced an ideotype in kabuli chickpea (*Cicer arietinum* L.) which is high-yielding, extra-large-seeded, and double- or multi-podded, has high plant height and imparipinnate-leafed traits, and is heat tolerant and resistant to ascochyta blight [*Ascochyta rabiei* (Pass.) Labr.], which causes considerable yield losses, via marker-assisted selection. F_3_ and F_4_ lines were evaluated for agro-morphological traits divided into six classes, namely, (i) imparipinnate-leafed and single-podded progeny, (ii) imparipinnate-leafed and double-podded progeny, (iii) imparipinnate-leafed and multi-podded progeny, (iv) unifoliolate-leafed and single-podded progeny, (v) unifoliolate-leafed and double-podded progeny, (vi) unifoliolate-leafed and multi-podded progeny. F_3:4_ lines having 100-seed weight ≥ 45 g and double- or multi-podded traits were additionally assessed for resistance to ascochyta blight using molecular markers including SCY17_590_ and CaETR-1. Superior lines having higher values than their best parents were determined for all studied traits indicating that economic and important traits including yield and seed size in chickpea could be improved by crossing suitable parents. Imparipinnate-leafed and multi-podded plants had not only the highest number of pods and seeds per plant but also the highest yield. On the other hand, imparipinnate-leafed and single podded progeny had the largest seed size, followed by imparipinnate-leafed and double-podded progeny. Multi-podded plants produced 23% more seed yield than that of single-podded plants, while multi-podded plants attained 7.6% more seed yield than that of double-podded plants. SCY17_590_ and CaETR-1 markers located on LG4 related to QTL_AR2_ and QTL_AR1_ were found in 14 lines among 152 F_3:4_ lines. Six superior lines were selected for being double- or multi-podded, imparipinnate-leafed, suitable for combine harvest, heat-tolerant, and resistant to ascochyta blight, and having both of two resistance markers and extra-large seeds as high as 50–60 g per 100-seed weight. Resistance alleles from two different backgrounds for resistance to ascochyta blight were integrated with double- or multi-podded kabuli chickpea lines having high yield, extra-large seeds, high plant height, imparipinnate-leaves and high heat tolerance, playing a crucial role for future demands of population and food security. These approaches seem to be applicable in ideotype breeding for other important crop plants.

## Introduction

Chickpea is a valuable plant, providing nutritious food for the growing world population, and will become increasingly important with climate change due to its natural drought and heat tolerance ability. At the same time, it is the most important food legume cultivated among cool season food legumes in the arid and semi-arid regions of the world under rainfed conditions. Globally, it was cultivated on 13.7 Mha with an annual production of 14.2 million tons^−1^, and had a trade volume of $ 2.7 billion in 2019^1^. Cultivated chickpeas are divided into two main types as “desi” and “kabuli”^2^. The “desi” types have pigmented vegetative parts and pink flowers, and the seeds are generally small and colored (mostly dark) with a thick seed coat. The “desi” chickpeas occupy about 80–85% of the chickpea cultivation areas in the world and are mainly grown in South Asia, East Africa and Australia^3^. The “kabuli” types have non-pigmented vegetative parts, white flowers and relatively large, cream-colored seeds with a thin seed coat and are mostly cultivated in the Mediterranean Basin, the Near East and East Asia^4^.

The term ideotype was first used as ‘idiotyp-’ by Siemens^5^ and defined as the sum total of hereditary objects in an organism, containing chromosomal genes and extra-chromosomal genes^6^. The concept of ‘crop ideotype’ was first introduced in cereals at the end of the 1960s by Donald^7^. As a concept, a crop ideotype is coined as a biological plant model expected to better perform for seed yield, oils and other useful characteristics when developed as a cultivar^7^. After cereals^8–10^, breeding for crop ideotype has enormously impacted on breeding programs and breeders^11^ resulting in considerable yield increases of agricultural crops from 1961 to the present date^1^. The ideotype description for chickpea (*Cicer arietinum* L.) was introduced in the late 1970s by Bahl and Jain^12^ and it started to become widely popular in later years^13–17^. The ideotype chickpeas are said to differ from eco-geological region to region in which they are produced in the world, based on agronomical, morphological, physiological and phenological characteristics^14,15,17–21^. In the mentioned ideotype chickpeas, requirements of farmers or producers and consumers have been hardly taken into consideration by breeders. Considering the shortcomings in the previously introduced ideotype chickpeas, more universal ideotype chickpeas were suggested with tolerable reactions to a/biotic stresses in the production areas and with acceptable morphological and physiological properties within certain limits. Farmers’ requirements were listed as high and stable seed yield, resistance to local diseases and pests, high N_2_ fixation capacity, extra-large-seeded “kabuli” chickpeas for some regions of the world, iron-deficiency-resistant, suitable plant height for combine harvest, and herbicide-resistant^22^.

Once breeding for crop ideotype is imagined by plant breeders for their own needs, the challenge becomes the creation of an ideotype in a plant breeding program^23^. There are important morphological, physiological, phenological, technological and agricultural traits in ideotype chickpeas. One of these is undoubtedly plant height, which is extremely important in terms of mechanized harvest^22,24,25^. The most important of the phenological features is earliness, which plays a crucial role in getting rid of drought and high/low temperature stresses^22,26–28^ forecast to increase in the near future due to global warming especially in areas where chickpeas are grown under rainfed conditions^29,30^. Extra-large seeds in “kabuli” chickpeas are attractive to consumers so producers are forced to produce them due to selling them at a high price both in domestic and international markets^31–34^. In addition to the high price of extra-large-seeded “kabuli” chickpeas, they produced better vigor than those of their counterparts during the seedling stage^22^. The main determinants of seed yield in chickpeas are biological yield, harvest index, number of pods per plant and 100-seed weight^35,36^. It is desired that yield and yield components such as biological yield, harvest index, number of pods per plant and 100-seed weight are as high as possible. Almost all chickpeas cultivated in the world are in the form of imparipinnate (compound) leaves^32,37^, and although a few chickpea cultivars have recently been introduced into agriculture with unifoliolate (simple) leaves^38^, it has been determined that chickpeas with imparipinnate leaves have more photosynthetic area than that of unifoliolate leaves^32,39,40^. Another desirable morphological trait can be listed as double pods per axil because these chickpeas produce more seed yield^41–45^ and better stability than that of single-podded ones^46,47^. Additionally, this has been reported in triple and multi-flowered chickpeas but the effect on yield has not been studied so far. Considering chickpea diseases, undoubtedly one of the most important chickpea diseases in the world is ascochyta blight caused by *Ascochyta rabiei* (Pass.) Labr. It reduces the yield by 100% under suitable conditions^48,49^. Blight disease resistance genes have been transferred from resistance resources^50–52^. However, there is a need to combine resistance genes into a chickpea genotype.

The Covid-19 epidemic experienced around the world for about 2 years and the global warming caused by climate change^53^ have revealed a gap between food demand and food supply^54^. The biggest challenge for plant breeders today is not only to meet the need for food due to climate change, but also to meet the future food needs of increasing population growth^55,56^. In this sense, it is imperative to increase the yield in edible legumes, especially for chickpea plants. The aims of the present study were to integrate resistance alleles from two different backgrounds for resistance to ascochyta blight with high-yield, extra-large-seeded, double- or multi-podded, high-plant-height, heat-tolerant and imparipinnate-leafed traits in kabuli chickpea under heat stress conditions.

## Results

### Qualitative morphological traits

Plants were segregated as imparipinnate leaf and single-pod, imparipinnate leaf and double-pods, imparipinnate leaf and multi-pods, unifoliolate leaf and single-pod, unifoliolate leaf and double-pods and unifoliolate leaf and multi-pods (Tables 1, 2).

**Table 1:** Means ± standard errors and range for agro-morphological traits in F_3_ population derived from intraspecific crosses between Sierra (single-podded and unifoliolate-leafed) and CA 2969 (double-podded and imparipinnate-leafed). I–S: imparipinnate-leafed and single-podded progeny, I–D: imparipinnate-leafed and double-podded progeny, I–M: imparipinnate-leafed and multi-podded progeny, U–S: unifoliolate-leafed and single-podded progeny, U–D: unifoliolate-leafed and double-podded progeny, U–M: unifoliolate-leafed and multi-podded progeny.

Traits	Sierra (♀)	CA 2969 (♂)	I–S	I–D	I–M	U–S	U–D	U–M	F_3_	
	${\overline{\text{X}}} \pm {\text{S}}_{{{\overline{\text{X}}}}}$	${\overline{\text{X}}} \pm {\text{S}}_{{{\overline{\text{X}}}}}$	${\overline{\text{X}}} \pm {\text{S}}_{{{\overline{\text{X}}}}}$	${\overline{\text{X}}} \pm {\text{S}}_{{{\overline{\text{X}}}}}$	${\overline{\text{X}}} \pm {\text{S}}_{{{\overline{\text{X}}}}}$	${\overline{\text{X}}} \pm {\text{S}}_{{{\overline{\text{X}}}}}$	${\overline{\text{X}}} \pm {\text{S}}_{{{\overline{\text{X}}}}}$	${\overline{\text{X}}} \pm {\text{S}}_{{{\overline{\text{X}}}}}$	${\overline{\text{X}}} \pm {\text{S}}_{{{\overline{\text{X}}}}}$	Range
Days to first flowering (days)	48.3 ± 0.08	50.0 ± 0.50	47.1 ± 0.23	47.2 ± 0.19	47.3 ± 0.31	45.9 ± 0.30	47.6 ± 0.32	46.6 ± 0.46	46.9 ± 0.12	37–76
Days to 50% flowering (days)	50.3 ± 0.08	52.3 ± 0.42	49.9 ± 0.20	50.2 ± 0.17	50.1 ± 0.28	48.9 ± 0.23	50.4 ± 0.25	49.7 ± 0.38	49.9 ± 0.10	39–73
Plant height (cm)	52.3 ± 0.61	42.7 ± 0.08	43.8 ± 0.27	44.4 ± 0.25	46.1 ± 0.73	48.8 ± 0.41	49.8 ± 0.53	53.3 ± 0.72	46.3 ± 0.18	19–68
Main stems per plant (no)	2.3 ± 0.09	2.3 ± 0.08	2.7 ± 0.04	2.8 ± 0.04	3.1 ± 0.14	2.8 ± 0.05	2.9 ± 0.06	3.0 ± 0.08	2.8 ± 0.02	1–6
Canopy width (cm)	49.0 ± 1.34	58.3 ± 2.08	60.7 ± 0.76	63.8 ± 0.72	69.0 ± 2.13	51.7 ± 0.79	51.35 ± 0.82	53.8 ± 1.39	58.5 ± 0.40	10.0–96.0
First pod height (cm)	31.3 ± 0.38	33.3 ± 0.79	29.9 ± 0.26	30.7 ± 0.25	31.7 ± 0.92	31.6 ± 0.41	32.2 ± 0.43	33.0 ± 0.70	30.9 ± 0.15	13–46
Pods per plant (no)	36.7 ± 2.05	42.7 ± 2.7	62.6 ± 1.87	69.5 ± 1.92	74.7 ± 6.08	48.6 ± 1.75	46.5 ± 1.62	49.1 ± 2.61	59.5 ± 0.96	1–254
Seeds per plant (no)	32.7 ± 2.02	50.7 ± 2.46	67.7 ± 2.07	76.9 ± 2.14	92.4 ± 8.00	51.5 ± 1.86	50.9 ± 1.49	54.4 ± 3.02	64.9 ± 1.07	1–267
Seed yield (g)	15.2 ± 0.86	13.9 ± 0.72	24.3 ± 0.74	26.2 ± 0.71	29.9 ± 2.68	16.3 ± 0.58	15.7 ± 0.55	16.2 ± 0.85	21.9 ± 0.37	1–79.0
100-seed weight (g)	46.9 ± 0.31	27.4 ± 0.34	42.4 ± 0.38	41.4 ± 0.39	40.5 ± 1.48	38.5 ± 0.45	39.3 ± 0.47	37.7 ± 0.78	40.7 ± 0.21	7.0–64.0
Biological yield (g)	39.0 ± 2.01	29.5 ± 1.57	50.4 ± 1.37	54.1 ± 1.29	60.6 ± 4.35	38.7 ± 1.27	38.3 ± 1.18	39.0 ± 1.66	47.3 ± 0.68	4.1–147.0
Harvest index (%)	38.4 ± 0.27	47.5 ± 0.33	46.1 ± 0.59	46.8 ± 0.56	49.2 ± 2.22	41.1 ± 0.47	39.9 ± 0.51	40.4 ± 0.76	44.3 ± 0.29	0.4–62.4

**Table 2:** Means ± standard errors and range for agro-morphological traits in F_4_ population derived from intraspecific crosses between Sierra (single-podded and unifoliolate-leafed) and CA 2969 (double-podded and imparipinnate-leafed). I–S: imparipinnate-leafed and single-podded progeny, I–D: imparipinnate-leafed and double-podded progeny, I–M: imparipinnate-leafed and multi-podded progeny, U–S: unifoliolate-leafed and single-podded progeny, U–D: unifoliolate-leafed and double-podded progeny, U–M: unifoliolate-leafed and multi-podded progeny.

Traits	Sierra (♀)	CA 2969 (♂)	I–S	I–D	I–M	U–S	U–D	U–M	F_4_	
	${\overline{\text{X}}} \pm {\text{S}}_{{{\overline{\text{X}}}}}$	${\overline{\text{X}}} \pm {\text{S}}_{{{\overline{\text{X}}}}}$	${\overline{\text{X}}} \pm {\text{S}}_{{{\overline{\text{X}}}}}$	${\overline{\text{X}}} \pm {\text{S}}_{{{\overline{\text{X}}}}}$	${\overline{\text{X}}} \pm {\text{S}}_{{{\overline{\text{X}}}}}$	${\overline{\text{X}}} \pm {\text{S}}_{{{\overline{\text{X}}}}}$	${\overline{\text{X}}} \pm {\text{S}}_{{{\overline{\text{X}}}}}$	${\overline{\text{X}}} \pm {\text{S}}_{{{\overline{\text{X}}}}}$	${\overline{\text{X}}} \pm {\text{S}}_{{{\overline{\text{X}}}}}$	Range
Days to first flowering	56.4 ± 0.14	59.5 ± 0.16	63.1 ± 0.52	65.6 ± 0.36	62.0 ± 0.88	61.8 ± 0.67	62.2 ± 0.52	62.2 ± 0.76	62.5 ± 0.23	55–88
Days to 50% flowering	62.4 ± 0.13	66.4 ± 0.15	68.0 ± 0.50	67.7 ± 0.31	67.4 ± 0.67	67.6 ± 0.59	67.6 ± 0.46	67.3 ± 0.70	67.7 ± 0.20	61–92
Plant height (cm)	56.8 ± 0.33	57.3 ± 0.36	50.9 ± 0.68	52.3 ± 0.44	53.2 ± 1.97	52.4 ± 0.92	57.7 ± 0.76	56.1 ± 1.22	53.3 ± 0.32	30–71
Main stems per plant (no)	3.4 ± 0.13	4.3 ± 0.14	4.7 ± 0.16	4.7 ± 0.11	5.2 ± 0.60	4.4 ± 0.15	4.6 ± 0.13	4.9 ± 0.24	4.6 ± 0.07	1–10
First pod height (cm)	43.1 ± 0.60	40.0 ± 0.62	28.4 ± 0.54	29.7 ± 0.38	31.1 ± 1.49	32.7 ± 0.70	35.6 ± 0.70	33.9 ± 0.73	31.3 ± 0.28	17–56
Canopy width (cm)	43.8 ± 0.47	55.7 ± 0.49	52.9 ± 1.20	54.6 ± 0.86	55.7 ± 3.63	45.0 ± 1.16	47.0 ± 0.92	46.9 ± 1.59	50.9 ± 0.52	10.0–84.0
Pods per plant (no)	11.6 ± 1.21	32.5 ± 4.39	40.7 ± 2.34	46.5 ± 1.94	46.9 ± 13.12	31.9 ± 1.96	32.6 ± 1.80	32.1 ± 2.94	39.5 ± 1.06	1–126
Seeds per plant (no)	11.4 ± 1.23	42.0 ± 5.92	43.3 ± 2.57	48.7 ± 2.16	54.4 ± 15.92	33.3 ± 2.18	31.8 ± 1.93	35.9 ± 3.54	41.4 ± 1.19	1–133
Seed yield (g)	5.3 ± 0.57	12.0 ± 1.64	16.9 ± 1.00	18.1 ± 0.77	19.6 ± 5.13	11.9 ± 0.78	11.8 ± 0.67	11.8 ± 1.01	15.3 ± 0.43	0.1–51.2
100-seed weight (g)	46.3 ± 1.03	28.9 ± 0.50	40.7 ± 0.95	39.8 ± 0.74	44.6 ± 4.18	36.7 ± 0.96	39.1 ± 1.04	36.8 ± 1.82	39.3 ± 0.45	10.0–69.6
Biological yield (g)	16.6 ± 1.26	26.7 ± 3.52	42.9 ± 2.34	47.3 ± 1.68	43.3 ± 9.87	36.0 ± 2.30	38.2 ± 1.80	33.8 ± 2.86	42.0 ± 0.98	0.3–110.4
Harvest index (%)	30.9 ± 1.45	44.4 ± 0.49	38.5 ± 1.08	37.3 ± 0.84	43.5 ± 2.41	32.8 ± 1.10	30.7 ± 0.97	36.0 ± 1.55	35.7 ± 0.49	0.8–59.3

### Transgressive segregations and superior lines for agro-morphological traits in the F_3_

**Figure 1:**
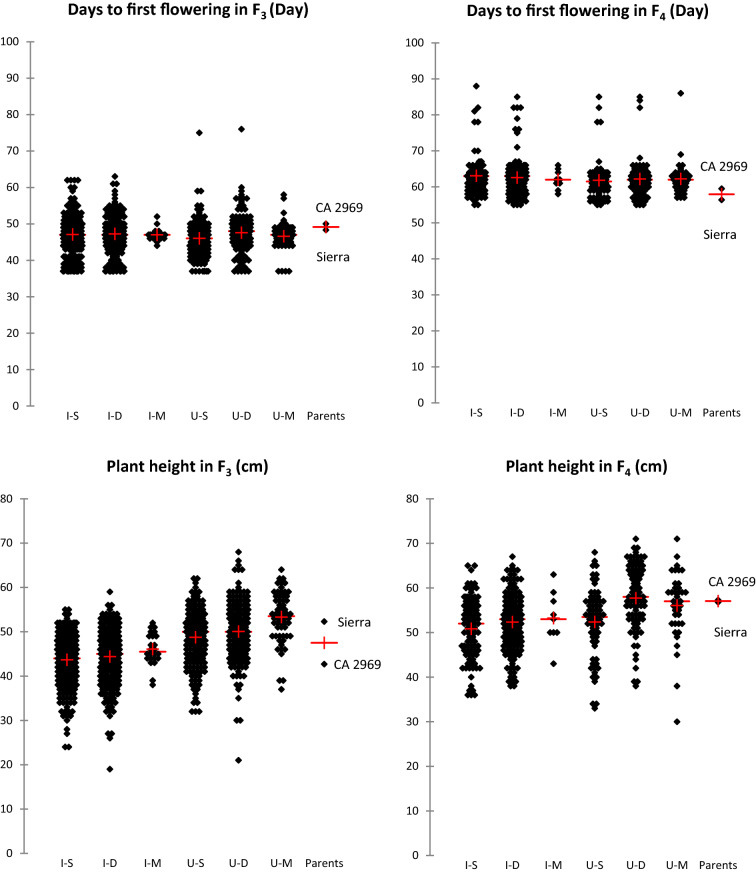
Means (+) of transgressive segregations and superior lines for days to first flowering and plant height in F_3_ and F_4_ populations derived from intraspecific crosses between Sierra and CA 2969. Each dot represents a progeny. I–S: imparipinnate-leafed and single-podded progeny, I–D: imparipinnate-leafed and double-podded progeny, I–M: imparipinnate-leafed and multi-podded progeny, U–S: unifoliolate-leafed and single-podded progeny, U–D: unifoliolate-leafed and double-podded progeny, U–M: unifoliolate-leafed and multi-podded progeny.

**Figure 2:**
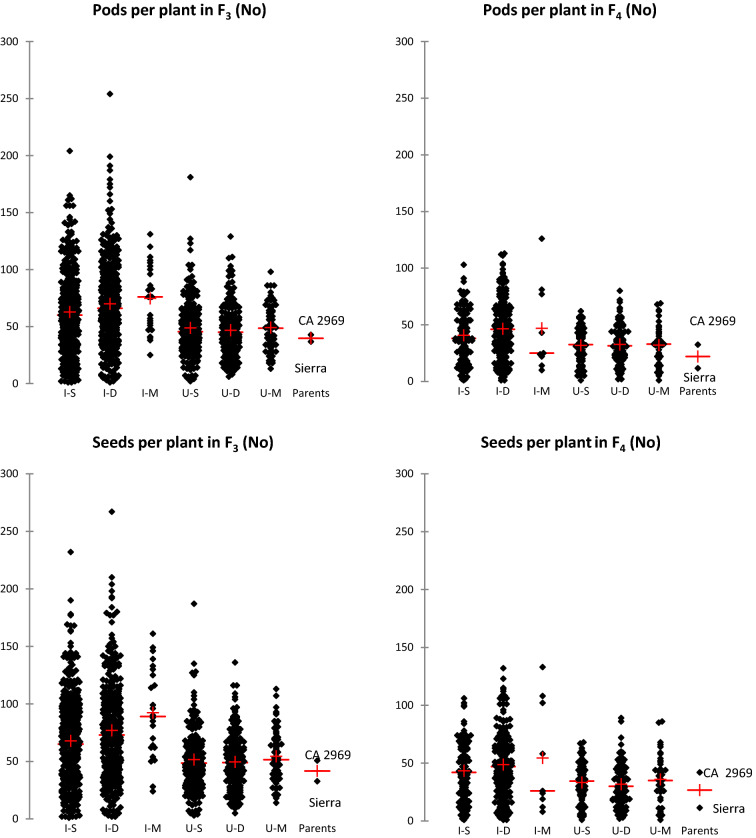
Means (+) of transgressive segregations and superior lines for pods and seeds per plant in F_3_ and F_4_ populations derived from intraspecific crosses between Sierra and CA 2969. Each dot represents a progeny. I–S: imparipinnate-leafed and single-podded progeny, I–D: imparipinnate-leafed and double-podded progeny, I–M: imparipinnate-leafed and multi-podded progeny, U–S: unifoliolate-leafed and single-podded progeny, U–D: unifoliolate-leafed and double-podded progeny, U–M: unifoliolate-leafed and multi-podded progeny.

**Figure 3:**
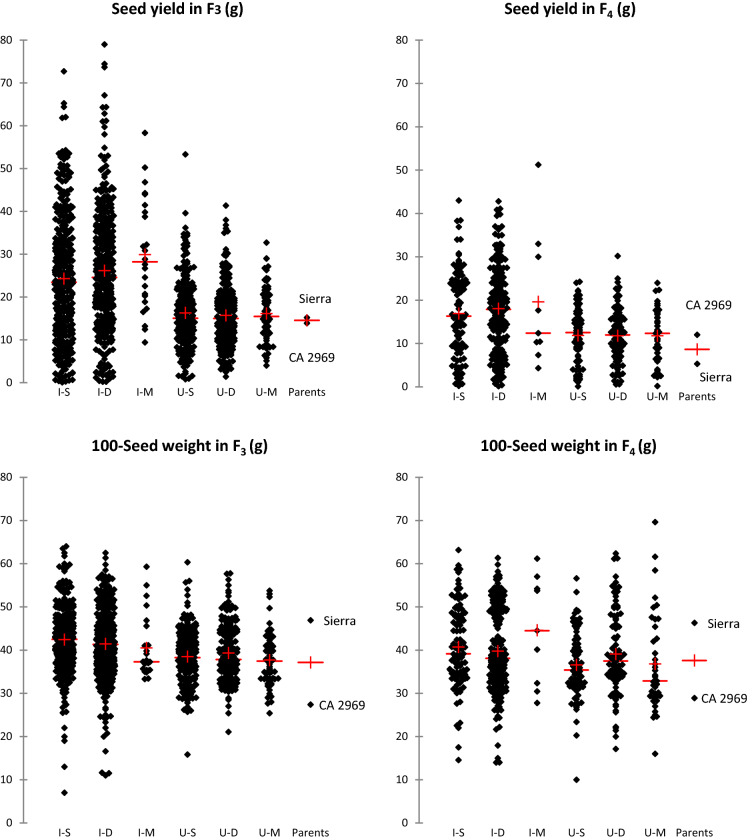
Means (+) of transgressive segregations and superior lines for seed yield per plant and 100-seed weight in F_3_ and F_4_ populations derived from intraspecific crosses between Sierra and CA 2969. Each dot represents a progeny. I–S: imparipinnate-leafed and single-podded progeny, I–D: imparipinnate-leafed and double-podded progeny, I–M: imparipinnate-leafed and multi-podded progeny, U–S: unifoliolate-leafed and single-podded progeny, U–D: unifoliolate-leafed and double-podded progeny, U–M: unifoliolate-leafed and multi-podded progeny.

According to the data analyses on descriptive statistics of the F_3_ population, transgressive segregations were determined for all agro-morphological traits including 100-seed weight (Table 1). Minimum and maximum values of days to first flowering of F_3_ population were found to be 37 and 76 days, respectively, whereas days to 50% flowering in F_3_ population ranged from 39 to 73 days, respectively. Days to first flowering and days to 50% flowering of Sierra and CA 2969 were 48.3–50.3 days and 50–52.3 days, respectively. Plant height of the genotypes in F_3_ population varied from 19 to 68 cm, while the plant height of Sierra and CA 2969 was measured as 52.3 and 42.7 cm, respectively. The average first pod height in F_3_ population was 30.9 cm, while it was 31.3 cm for Sierra and 33.3 cm for CA 2969 (Table 1, Fig. 1). The number of main stems per plant in F_3_ population was 1–6, whereas it was 2–3 in the Sierra and CA 2969. The canopy width in F_3_ population varied between 10 and 96 cm, while it was 49 cm in the Sierra and 58.3 cm in the CA 2969. The number of seeds per plant in F_3_ population was 1–267 and the average of this trait was recorded as 32.7 and 50.7 in the Sierra and CA 2969, respectively (Table 1, Fig. 2). Seed yield per plant ranged from 1 to 79 g in F_3_ population, while mean seed yield per plant was 15.2 and 13.9 g in the Sierra and CA 2969, respectively. The 100-seed weight ranged from 7 to 64 g in F_3_ population, while the 100-seed weight was recorded as 46.9 g and 27.4 g in the Sierra and CA 2969, respectively. Biological yield in F_3_ population was determined as 4–147 g, while harvest index was 0.4–62.4%. Biological yield and harvest index in Sierra were determined as 39 g and 38.4%, while in CA 2969 they were calculated as 29.5 g and 47.5%, respectively (Table 1, Fig. 3).

### Transgressive segregations and superior lines for agro-morphological traits in the F_4_

Minimum and maximum values of days to first flowering in F_4_ were found to be 55 and 88 days, respectively, whereas days to 50% flowering in F_4_ ranged from 61 to 92 days, respectively (Table 2). Days to first flowering and days to 50% flowering of Sierra and CA 2969 were 56.4–62.4 days and 59.5–66.4 days, respectively. Plant height in F_4_ varied from 30 and 71 cm, while the plant heights of Sierra and CA 2969 were 56.8 and 57.3 cm, respectively (Table 2, Fig. 1). The average first pod height in F_4_ was 31.3 cm, while it was 43.1 cm for Sierra and 40 cm for CA 2969. The number of main stems per plant in F_4_ was 1–10, whereas it was 3.4 in the Sierra and 4.3 in the CA 2969. The canopy width in F_4_ varied between 10 and 84 cm, while it was 43.8 cm in the Sierra and 55.7 cm in the CA 2969. The number of seeds per plant in F_4_ was 1–133 and the average of this trait was 11.4 and 42 in the Sierra and CA 2969, respectively (Table 2, Fig. 2). Seed yield per plant ranged from 0.1 to 51.2 g in F_4_, while mean seed yield per plant was 5.3 and 12 g in the Sierra and CA 2969, respectively. The 100-seed weight ranged from 10 to 69.6 g in F_4_, while the 100-seed weight was 46.3 g and 28.9 g in the Sierra and CA 2969, respectively. Biological yield in F_4_ was 0.3–110.4 g, while harvest index was 0.8–59.3%. Biological yield and harvest index were 39 g and 38% in Sierra, while in CA 2969, they were 30 g and 48%, respectively (Table 2, Fig. 3).

### Superior lines for agro-morphological traits in the F_3:4_

As for the selected F_3:4_ superior lines for 100-seed weight and double/multi-podded traits, transgressive segregations for all agro-morphological traits are shown in Table 3. Days to first flowering and 50% flowering were 43–66 days and 59–74 days, respectively. Plant height and number of main stems per plant of the F_3:4_ superior lines varied from 39 to 73 cm and from 2 to 6, respectively (Table 3). Average first pod height was 41.2 cm, while mean canopy width was 50.1 cm. Pods and seeds per plant ranged from 1 to 76 and from 1 to 75, respectively. Seed yield per plant varied from 0.4 to 30.7 g. 100-seed weight ranged from 23.2 to 69, while mean seed yield per plant was 43.8 g. Biological yield in F_3:4_ superior lines was determined as 0.5–74.1 g, while harvest index was 3.5–86.5%.

**Table 3:** Means ± standard errors and range for agro-morphological traits in F_3:4_ superior lines having 100-seed weight ≥ 45 g and double/multi-podded selected in F_3_. I–D/M: imparipinnate-leafed and double/multi-podded progeny, U–D/M: unifoliolate-leafed and double/multi-podded progeny.

Traits	Sierra	CA 2969	F_3:4_		I–D/M	U–D/M
	${\overline{\text{X}}} \pm {\text{S}}_{{{\overline{\text{X}}}}}$	${\overline{\text{X}}} \pm {\text{S}}_{{{\overline{\text{X}}}}}$	${\overline{\text{X}}} \pm {\text{S}}_{{{\overline{\text{X}}}}}$	Range	${\overline{\text{X}}} \pm {\text{S}}_{{{\overline{\text{X}}}}}$	${\overline{\text{X}}} \pm {\text{S}}_{{{\overline{\text{X}}}}}$
Days to first flowering	56.4 ± 0.14	59.5 ± 0.16	56.7 ± 0.10	43–66	57.1 ± 0.12	55.8 ± 0.20
Days to 50% flowering	62.4 ± 0.13	66.4 ± 0.15	65.2 ± 0.05	59–74	65.3 ± 0.07	65.2 ± 0.10
Plant height (cm)	56.8 ± 0.33	57.3 ± 0.36	59.6 ± 0.15	39–73	58.1 ± 0.18	62.8 ± 0.26
Main stems per plant (no)	3.4 ± 0.13	4.3 ± 0.14	3.6 ± 0.02	2–6	3.5 ± 0.02	3.6 ± 0.03
First pod height (cm)	43.1 ± 0.60	40.0 ± 0.62	41.2 ± 0.13	24–56	39.9 ± 0.15	43.7 ± 0.22
Canopy width	43.8 ± 0.47	55.7 ± 0.49	50.1 ± 0.12	30.0–65.0	51.1 ± 0.15	48.1 ± 0.19
Pods per plant (no)	11.6 ± 1.21	32.5 ± 4.39	17.7 ± 0.23	1–76	18.9 ± 0.30	15.2 ± 0.33
Seeds per plant (no)	11.4 ± 1.23	42.0 ± 5.92	16.5 ± 0.23	1–75	18.1 ± 0.31	13.5 ± 0.31
Seed yield (g)	5.3 ± 0.57	12.0 ± 1.64	7.0 ± 0.09	0.4–30.7	7.7 ± 0.12	5.7 ± 0.13
100-seed weight (g)	46.3 ± 1.03	28.9 ± 0.50	43.8 ± 0.13	23.2–69.0	44.0 ± 0.16	43.2 ± 0.24
Biological yield (g)	16.6 ± 1.26	26.7 ± 3.52	20.4 ± 0.22	0.5–74.1	20.4 ± 0.27	20.6 ± 0.39
Harvest index (%)	30.9 ± 1.45	44.4 ± 0.49	34.4 ± 0.25	3.5–86.5	37.6 ± 0.29	28.1 ± 0.36

### 100-seed weight in the F_3_ and F_4_ populations

**Figure 4:**
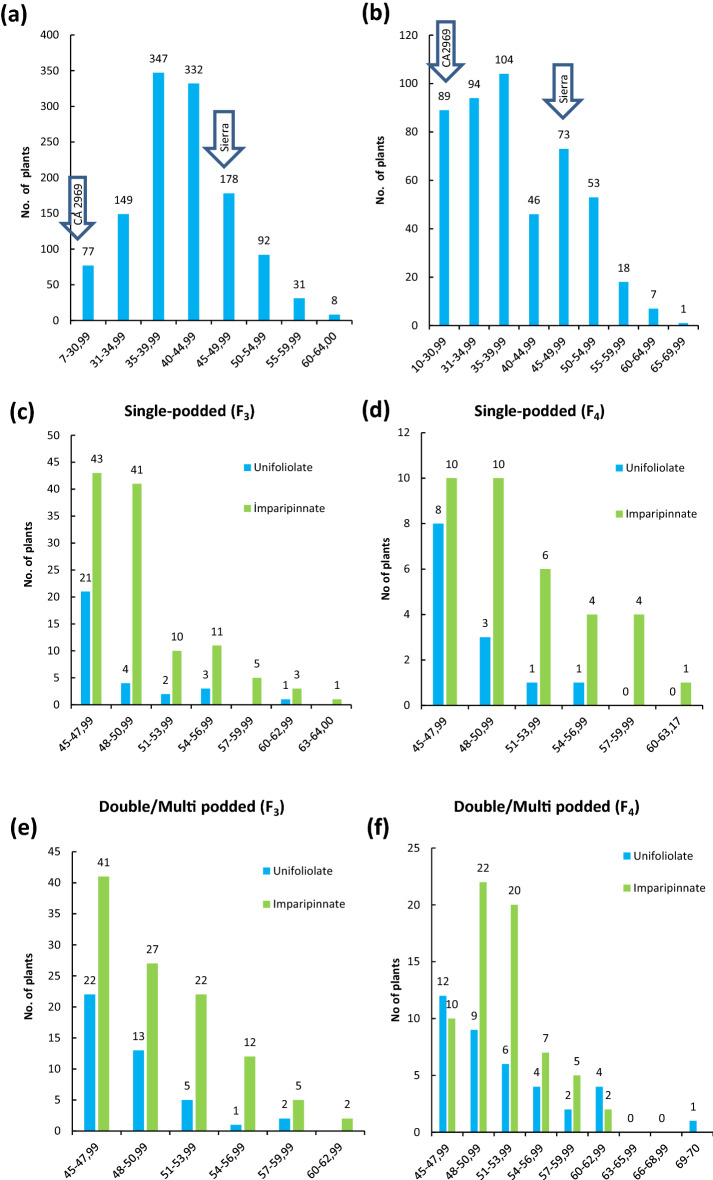

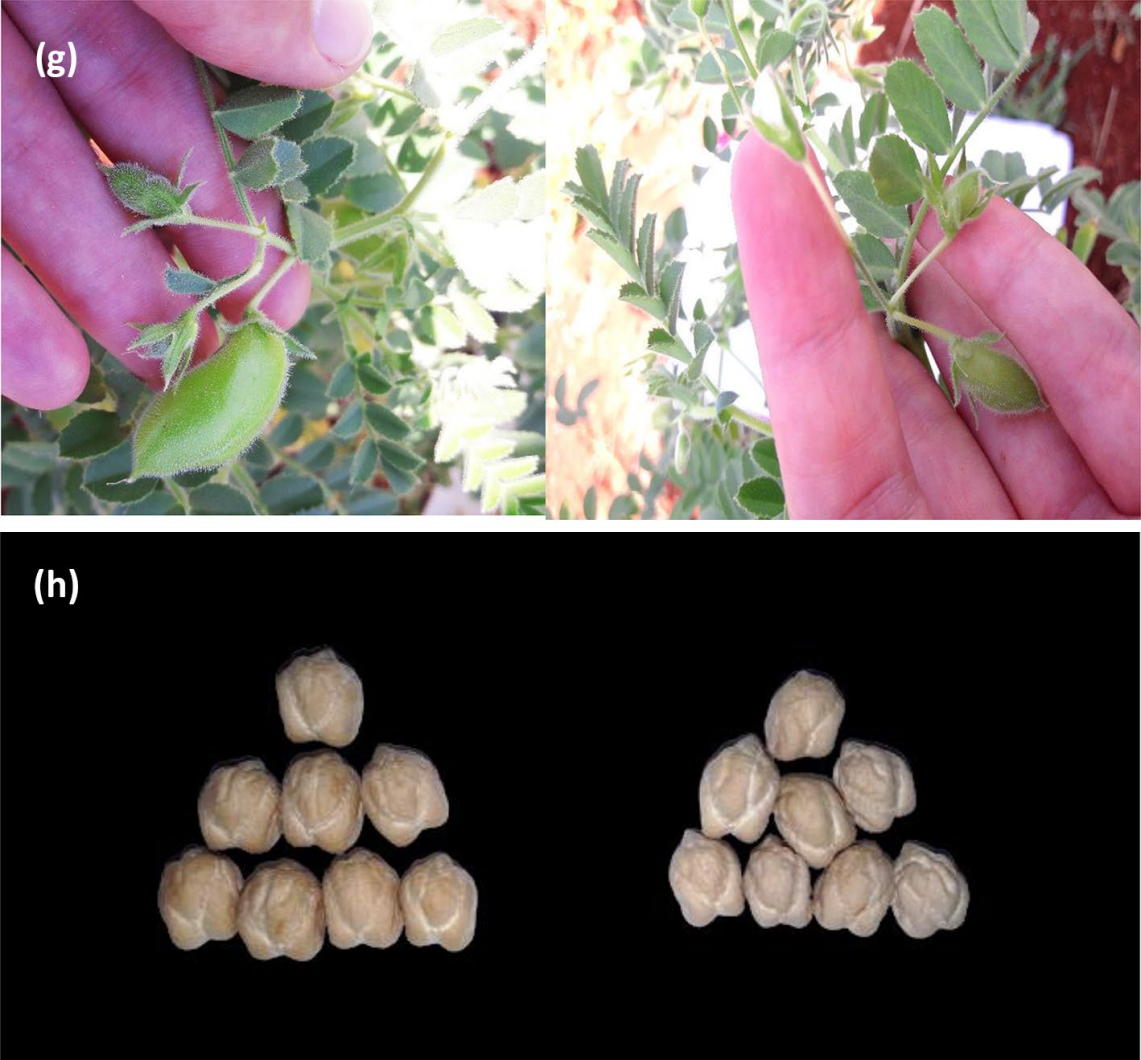
Distribution of 100-seed weight in F_3_ (**a**) and F_4_ (**b**). Distribution of 100-seed weight of genotypes with single-poddded and 100-seed weight ≥ 45 g according to leaf shape in F_3_ (**c**) and F_4_ (**d**). Distribution of 100-seed weight of genotypes with double/multi-poddded and 100-seed weight ≥ 45 g according to leaf shape in F_3_ (**e**) and F_4_ (**f**). A line in F_4_ (multi-podded per axil, imparipinnate-leafed) derived from intraspecific crosses between Sierra (single-podded and unifoliolate-leafed) and CA 2969 (double-podded and imparipinnate-leafed) (**g**). Seeds of a superior line (multi-podded, unifoliolate-leafed and 69.6 g per 100-seeds, left side) in F_4_ and its large-seeded parent Sierra (single-podded, unifoliolate-leafed and 46.9 g per 100-seeds, right side) (**h**).

The F_3_ and F_4_ populations were evaluated for seed size based on 100-seed weights and transgressive segregations were observed (Fig. 4a,b). In F_3_ and F_4_, a large number of genotypes had 100-seed weight ≥ 45 g (as selection criteria) and were larger than the parent Sierra (46.9 g) (Fig. 4c). Distribution of 100-seed weight depending on the pods per axil (single, double or multi podded) and leaf shapes (unifoliolate or imparipinnate) of genotypes with 100-seed weight ≥ 45 g is given in Fig. 4c–f. In single and double/multi-podded genotypes, a larger number of extra-large-seeded chickpeas (as large as Sierra) were available in more imparipinnate leaves than unifoliolate leaves, in both F_3_ and F_4_ (Fig. 4c–f). In F_3_, 24 single-podded genotypes and 22 double/multi-podded genotypes had 100-seed weight ≥ 54 g and among them, the highest value was 64 g in an imparipinnate-leafed and single-podded genotype (Fig. 4c,e). In F_4_, 10 single-podded genotypes and 25 double/multi-podded genotypes had 100-seed weight ≥ 54 g (Fig. 4d,f) and among them, the largest seed was 69.6 g in a unifoliolate-leafed and multi-podded genotype (Fig. 4f,h). The selection was performed from F_3_ to F_3:4_ generations based on 100-seed weight ≥ 45 g in double/multi-podded genotypes and 152 F_3:4_ lines were selected.

### Selection for resistance to ascochyta blight in double/multi-podded and extra-large seeded F_3:4_ lines

**Figure 5:**
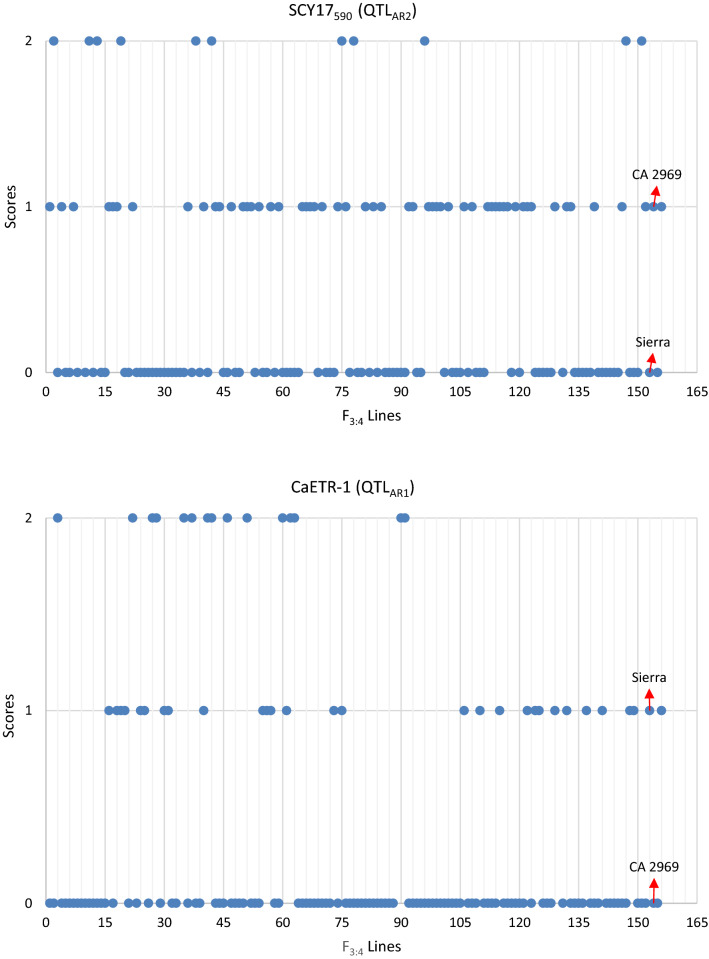
Distribution of scores according to SCY17_590_ and CaETR-1 markers associated with resistance to ascochyta blight in 152 F_3:4_ lines. (0: Homozygous susceptible, 1: Homozygous resistant, 2: Heterozygous).

The parents, 152 F_3:4_ lines, susceptible (ILC 1929) and resistant (ILC 3279) controls were genotyped for the markers SCY17_590_ (linked to QTL_AR2_) and CaETR-1 (linked to QTL_AR1_). ILC 3279 and ILC 1929 had resistant and susceptible alleles for both markers, respectively (Supplementary Table S1, Fig. 5). CA 2969 and Sierra possessed the resistant and susceptible allele for the SCY17_590_, respectively, while CA 2969 and Sierra had susceptible and resistant alleles for the CaETR-1, respectively. In the 152 F_3:4_ lines, numbers of the resistant: heterozygous: susceptible lines for the SCY17_590_ and CaETR-1 markers were found to be 54:11:85 (no amplification in two F_3:4_ lines) and 27:15:105 (no amplification in three F_3:4_ lines), respectively (Supplementary Table S1 and Fig. 5). A total of 14 F_3:4_ lines had both QTLs associated with blight resistance (Supplementary Table S1). Nine of them had homozygous resistant alleles in both markers. In the remaining five lines, two lines were heterozygous and homozygous for QTL_AR2_ and QTL_AR1_, respectively. Two other lines were homozygous and heterozygous for QTL_AR2_ and QTL_AR1_, respectively, while one line was heterozygous for both QTLs. Six lines having 100-seed weight ≥ 50 g had resistant alleles for both QTLs and among them, the highest 100-seed weight was 59.8 g in an imparipinnate-leafed and double-podded line. On the other hand, 100-seed weight of a line found to be resistant only in SCY17_590_ marker was determined as 62.5 g (Supplementary Table S1).

### Heat tolerance

**Figure 6:**
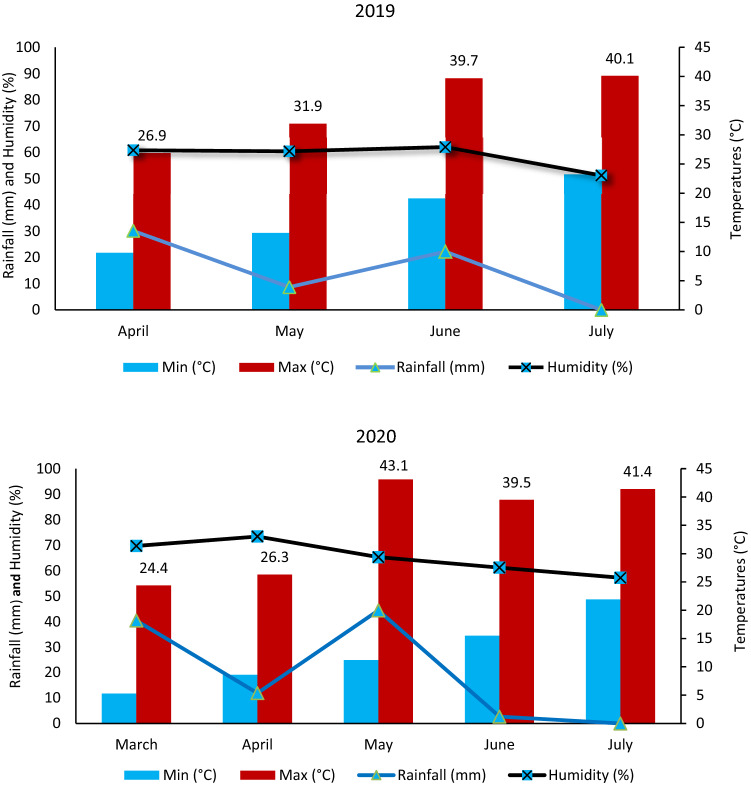
Monthly minimum and maximum temperatures, rainfall and relative humidity from sowing to harvest time in 2019 and 2020 growing season.

Plants were subjected to maximum temperatures over 30 °C during flowering in the first year, while they were exposed to heat stress during the pod setting stage of as high as about 40 °C in the first year. In the second year, plants tried to keep standing with heat stress of 43.1 °C during flowering and about 39.5 °C during pod setting (Fig. 6).

### Integrated traits

Six superior lines that were double- or multi-podded, imparipinnate-leafed, suitable for combine harvest, and heat-tolerant, and that had extra-large seeds as high as 50–60 g per 100-seed weight were integrated with resistance to ascochyta blight having both of two resistance markers (Tables 1, 2, 3, Fig. 5, Supplementary Table S1).

## Discussion

Double flowers or pods per axil were governed by a recessive single gene^14,42,44,47^, while imparipinnate leaf shape in cultivated chickpea was controlled by a dominant gene^14,57–59^. In early generations such as F_2_ and F_3_, after the genotypes with double pods per axil trait are selected, as it is controlled by a single recessive gene^14^, this trait is not expected to segregate in subsequent generations.

Genetic studies have shown that transgressive segregations are mostly due to the occurrence of combinations of alleles from both parents with the same effect (complementary gene effect)^60,61^. It is possible that hybrid individuals combining the ‘desired’ or ‘unwanted’ alleles of both parents show superior (unexpected or extreme) phenotypes. Similarly, in this study, transgressive segregations were determined for all agro-morphological traits and 100-seed weight not only in F_3_ but also in F_4_ population (Tables 1, 2, 3, Figs. 1, 2, 3, 4). Among the progeny, 209 progeny in F_3_ population had higher 100-seed weight than that of the best parent Sierra with 46.9 g and of these, a total of 131 progeny in F_3_ population had higher 100-seed weight than 50 g (Fig. 4a). It was also determined that some of them could produce double pods and be larger seeded than expected up to 62.5 g (Fig. 4e). Transgressive segregations are considered to be due to the complementary effect of genes and activity of suppressed recessive genes in the parents and to occur especially in crosses with wild origin plants^35,45,60–62^. Moreover, additional explanations have been outlined in relation to observations of transgressive segregations in segregating generations (see detail in Rieseberg et al.^61^). These consist of: (i) an elevated mutation rate, (ii) reduced developmental stability; (iii) epistasis or non-additivity of allelic effects among loci; (iv) overdominance or non-additivity of allelic effects within a locus; (v) unmasking of recessive alleles that are heterozygous in the parents; (vi) different number of chromosomes; and (vii) complementary action of additive alleles that are dispersed between parents. As promising chickpea genotypes, extra-large seeded genotypes containing double or multiple pods were determined not only in F_3_ but also in F_4_ (Fig. 4e,f).

The double-podded trait in chickpeas is one of the important traits since it provides yield increase and yield stability compared to its single-podded counterparts^41–43,46,47^. While most cultivated chickpeas in the world have a single flower and therefore a single pod in the flower cluster^63,64^, double-podded chickpeas were first recorded by Khan and Akhtar^65^ and governed by a single recessive gene ‘*s*’ or ‘*sfl*’^14^. As a result of the epistatic effect of the double-podded trait on a different background, multiple-flowered (more than two flowers) or multiple-podded progeny were first observed in some genotypes in F_3_ and then F_4_ (Fig. 4g). Single-podded genotypes had larger seed size than double- and multi-podded genotypes, while double- and multi-podded genotypes had higher seed yield, numbers of pods and seeds per plant than single-podded genotypes. The triple-flowered trait (*sfl*^*t*^) is controlled by a single recessive gene in the cultivated chickpeas^66^. The double-flowered trait (*sfl*^*d*^) is dominant on the triple-flowered trait, and the dominance relationship of these alleles in this locus has been reported to be *sfl* (single flower) > *sfl*^*d*^ (double flower) > *sfl*^*t*^ (triple flower)^66^. In our study, the definition of ‘multi-flowered’ was used since the formation of four and five flowers per axil besides the formation of triple flowers was observed. When the genotypes with four and five flowers were examined, these formations were observed together with ‘triple flowers’. These findings are similar to the triple-multi-flowered phenotypes resulting from crossing of double-flowered and multi-flowered genotypes in the allelism study for the *sfl* and *cym* genes conducted by Srinivasan et al.^66^. Therefore, it was interpreted that the formation of four or five flowers may also be due to the presence of the *cym* (multi flowers) gene^66,67^. In addition, it was determined that multi-flowered gene was expressed more phenotypically in unifoliolate leaf genotypes when compared to imparipinnate leaf genotypes (Tables 1, 2, 3). There may be a relationship between multi-flowered and unifoliolate leaf traits. However, in the genotypes with unifoliolate leaf, it was determined that ‘the third flower did not fill in pods’, while it was observed that pod filling was proportionally higher in multi-flowered plants in genotypes having imparipinnate leaf (Fig. 4g). It was stated that greater photosynthetically leaf area in the genotypes with imparipinnate leaf could be the reason for higher seed yield^39^. Multi-podded plants had 23% and 7.6% more seed yield than that of single-podded and double-podded plants in F_4_ population (Table 2). The seed yield advantage of multi-podded plants was greater in F_3_ than in F_4_ population (Tables 1, 2). Cho et al.^68^ concluded that QTLs for 100-seed weight and number of seeds per plant were determined on LG 4. Also, these two traits were found to be associated negatively^68^.

Genotypes having imparipinnate leaf had a higher seed yield than genotypes with unifoliolate leaf (Tables 1, 2). This result bore a resemblance to the findings of Abbo et al.^39^ in chickpea having imparipinnate leaf. In addition, it was determined that genotypes having imparipinnate leaf had higher 100-seed weight, number of pods and seeds than genotypes having unifoliolate leaf (Tables 1, 2). Higher seed yield and larger seed size in genotypes having imparipinnate leaf compared to unifoliolate-leafed genotypes were considered to be due to larger photosynthetic area (Tables 1, 2, 3, Fig. 4). Abbo et al.^39^ stated that chickpeas reach higher leaf area indices in both low and high seeding densities in genotypes with imparipinnate leaf. Most chickpea cultivars have imparipinnate leaves and several leaf shape mutations (unifoliolate and multipinnate) are available in chickpea^59,69,70^. Leaf shapes in chickpea are governed by two genes (*ml* and *sl*) through complementary gene actions^58^. The *ml* gene is dominant (*ml* + *sl*/*.sl*) in the multipinnate leaf, whereas it is recessive (*ml.*/*ml.*) in the unifoliolate leaf and both the genes are dominant form (*ml* + *sl* + /*…*) in imparipinnate leaf. Imparipinnate leaf shape was dominant over all other leaf shapes^57,59,71^. The unifoliolate leaf trait was introduced in kabuli cultivars released by the USDA program^72,73^. Furthermore, the area of a single imparipinnate leaf during seed development is about 2 or 3 times larger than that of a single unifoliolate leaf^39^. Imparipinnate-leafed progeny attained 35% higher yield than unifoliolate-leafed progeny (Table 2).

In the chickpea trade, seed size and color characteristics are important criteria although its acceptability varies according to cultural preferences in different parts of the world. Large-seeded chickpeas are preferred by farmers to produce these chickpeas, as they are sold at high prices in local and international markets due to consumer preference^31,32,34,46,74–76^. Additionally, seed size is an important part of yield and adaptation^77^. This is also considered as an important factor for subsequent plant growth parameters, including germination, seedling vigor and seedling biomass^78,79^. Therefore, large seed size provides an advantage to cope with drought stress compared to small seeds when planted deeper into the soil^22,80^. In chickpeas, it is known that studies on genetic control of large seed size have been carried out since the 1950s^81–85^. Inheritance of seed size was demonstrated to be monogenic^86^ and polygenic^84,87–89^ through different studies. Although seed size in chickpeas has a high heritability^84,90–92^, it is not only affected by genetic factors but also by the environment^14,17,44,93^. In terms of seed size, many genotypes (209 genotypes) with larger seeds than large-seeded parents were determined (Tables 2, 3, Fig. 4). The additive gene effect has been reported for seed size in chickpeas^84,94^ and shows that selection for this trait will be effective in early generations. Seed size in chickpea was mapped using inter- and intraspecific recombinant inbred lines (RILs) and two quantitative trait loci (QTL) were located in linkage groups: L1, L2, L4, L5, L7 and L8^95–98^.

Resistance to ascochyta blight has been one of the most important aims of chickpea breeding programs. Ascochyta blight is the most devastating disease of chickpea, attacking all upper parts of the plant (stems, leaves, pods and seeds) and the loss of seed yield can reach up to 100% under epidemic conditions in many chickpea growing regions around the world^48,99,100^. The introduction of winter sowing in order to increase the yield in the Mediterranean basin^101^ has led to the need to develop varieties that are resistant to blight disease, which is one of the main factors that reduce the yield during this growing season. Resistance to ascochyta blight is a quantitative trait and numerous QTLs have been located on the chickpea genetic map^102–114^. As a requirement of conducting breeding studies on quantitative traits, the breeding process is complicated by trying to combine genes or QTLs in a new variety to increase the level and durability of resistance. Therefore, marker-assisted selection for ascochyta blight resistance has been successfully and effectively used in recent years to increase the selection efficiency in the breeding process^50,51,103,115–119^. In recent years, several cultivars having an acceptable resistance level have been developed^38,117,120–122^. However, a significant portion of these cultivated varieties have small to medium seed sizes and do not meet the demands of the markets where large and extra-large seeded chickpea varieties are preferred. Since extra-large seeded kabuli chickpeas can find buyers at higher prices in the market, there is a need to develop extra-large seeded white/white-cream colored chickpeas. Resistance sources to blight have been identified by ICARDA from chickpea lines with medium seed size^123^. Some of these lines were included in breeding programs with large-seeded chickpeas. However, this allowed the selection of medium-sized chickpeas that were determined to be resistant to blight disease^117^. Resistance to ascochyta blight in chickpea is known to be controlled by more than two genes with small genes having an additive effect^124^. Knowledge on ascochyta blight was updated six years ago by Sharma and Ghosh^49^ and about 50 markers/QTLs were listed. Madrid et al.^118^ reported that the phenotype of 36 of 40 resistant genotypes (90%) and 14 susceptible genotypes could be accurately predicted by using the markers CaETR and SCY17_590_. The same markers were effectively used by Bouhadida et al.^125^ to select genotypes resistant to blight. In the present study, both parents were selected as resistant to blight disease according to the evaluations in the local field conditions of the countries where they were registered.

Although chickpea is tolerant to drought and heat stresses, it will be forced to be exposed to higher temperatures and more drought conditions owing to global warming as a result of climate change^29,30^. In the present study, plants were subjected to high temperatures as high as 40 °C during flowering and pod setting stages (Fig. 6). Despite the mentioned high temperatures, plants had more seed yield in F_3_ than in F_4_ population (Tables 1, 2, Fig. 6), meaning that yield could be decreased in homozygosity. High plant height is an important morphological trait for mechanized harvest in chickpea^22,24,25^, and plant height of 50–60 cm is considered as necessary for combine harvesting. In the present study, plant height ranged from 58 cm in double/multiple-podded plants having imparipinnate leaf to 62 cm double/multiple-podded plants having unifoliolate leaf (Table 3) when we considered superior lines. Days to first flowering in these superior lines were recorded to be 57 days in imparipinnate-leafed and double/multi-podded lines and 55 days in unifoliolate-leafed and double/multi-podded lines (Table 3). In spite of a crucial escape mechanism of earliness for drought and heat stress conditions, tolerance is the other vital mechanism in chickpea for drought and heat stresses^22,26–28^. In the present study, plants had a sufficient tolerance mechanism for heat stresses since they were exposed to considerable heat stress (Fig. 6).

According to the findings, the following ideotype was defined in kabuli chickpea:

- It should have imparipinnate-leafed traits, because these have more photosynthetic area than unifoliolate leaf type, and imparipinnate-leafed chickpeas attained 35% more seed yield than that of unifoliolate-leafed chickpeas under heat stress conditions.
- It should be double/multi-podded, since these plants had the highest number of pods and seeds per plant and also the highest yield. Multi-podded plants not only produced 23% more seed yield than that of single-podded plants, but also, multi-podded plants produced 7.6% more seed yield than that of double-podded plants under heat stress conditions.
- It should be extra-large-seeded as large as 50–60 g per 100-seed weight, because extra-large seeds are preferred by consumers and producers due to their high price in national and international markets and advantage during germination, related with drought.
- It should have enough plant height for combine harvest. Superior lines had 58–62 cm plant height.
- It should be heat tolerant, since heat stress is forecast to increase in the near future due to global warming as a result of climate change. Superior lines had a six times higher seed yield that that of the best parent under heat stress conditions.
- It should carry different resistance genes or QTLs for resistance to ascochyta blight, which is one of the widespread diseases of chickpea in the world, as minor genes from different backgrounds provide durable resistance. Two resistance markers (SCY17_590_ and CaETR-1 markers) were integrated into six superior lines via marker-assisted selection.
- Yield increases on a single plant basis will lead to significant yield increases per hectare. Thus, millions of tons of productivity of chickpea can be achieved worldwide with the ideotype improvement under heat stress, playing a crucial role for future demands of population and food security.
- These approaches seem to be applicable in ideotype breeding for other important crop plants.

## Methods

### Plant materials

Sierra (PI 631078) was crossed with CA 2969 (PI 632396) and the traits of the parents were previously described^38,126^. Sierra and CA 2969 chickpea genotypes/cultivars were registered by USDA-ARS in cooperation with the Washington Agricultural Research Center, Pullman, WA and CIFA, Cordoba, Spain, respectively. The procurement of seeds of Sierra and CA 2969 used in the present study complies with relevant institutional, national, and international guidelines and legislation. From F_1_ to F_3_ population, advancement of the progeny was detailed by studying inheritance of large seed size and qualitative traits^32^. A total of 626 F_3_ and 485 F_4_ lines were used in the present study. Also, 152 F_3:4_ lines with double-/multi-podded and 100-seed weight over 45 g were evaluated by marker-assisted selection (MAS) for resistance to ascochyta blight. These 152 lines were independently evaluated for agro-morphological traits.

### Experimental area and seasons

The study was conducted in fields at Akdeniz University, Antalya, Turkey (30° 38′ E, 36° 53′ N, 51 m above sea level) for two years during the crop seasons of 2019 and 2020. Plants were sown in April 2019 and March 2020, and harvested in July in both years.

### Agronomic applications

F_3_ and F_4_ lines were grown as progeny rows, while the parent plants were grown as four replicates (10 plants in a row) in the spring of 2019 and 2020. The selected 152 F_3:4_ lines were grown to have 20 siblings from each line and distributed randomly into two blocks in the spring of 2020. Plants were sown in rows of 2 m length and spaced 50 cm apart between rows with a within-row plant spacing of 10 cm. Drip irrigation was used in both growing seasons. Weeds were plucked by hand during planting and before flowering. No fertilizer application was made.

### Soil properties

Soil in the experimental area was sampled between 0 and 30 cm and then analyzed to define the experimental soil characteristics. Some plant nutrient elements were found to be at an adequate level, while organic matter and nitrogen, iron and zinc levels were defined to be at a low level. Soil texture was loam with CaCO_3_ of 26.5%, whereas pH was high with 7.69.

### Weather conditions

Plants were grown in the April-July period of 2019 and March–July period of 2020. Climatic data for the periods were provided by the T.C. Ministry of Agriculture and Forestry 4th Regional Directorate of Meteorology (Fig. 6). During the periods, total precipitation was recorded as 61 mm in 2019 and 99.4 mm in 2020. The highest precipitation was recorded in April with 30.1 mm in 2019 and in March with 40.4 mm in 2020, while the lowest precipitation was recorded in July with 0.0 mm in both years. While the highest temperature during the flowering stage of the plants was 31.9 °C in May 2019 and 43.1 °C in May 2020, the highest temperature was recorded as 39.7 °C in 2019 and 39.5 °C in 2020 during the pod setting stage (Fig. 6).

### Agro-morphological data

Qualitative traits such as leaf shape (as unifoliate or imparipinnate) and flower/pod per peduncle (as single/double/multiple flowers/pods) were recorded for each parent and progeny. In addition to the qualitative traits, the following quantitative traits, namely, days to first flowering (day) and days to 50% flowering (day), plant height (cm), first pod height (cm), number of main stems per plant (no), canopy width (cm), number of pods per plant (no), number of seeds per plant (no), seed yield per plant (g), biological yield per plant (g), 100-seed weight per plant (g) and harvest index (%) were evaluated. Seed size that is 100-seed weight was determined by using the following formula^32^:

$$100-seed \; weight \left(\mathrm{g}\right)=(Total \; seed \; weight \; per \; plant (\mathrm{g})/Total \; number \; of \; seeds \; per \; plant)\times 100.$$

### Transgressive segregations and superior lines

Transgressive segregation was coined as the occurrence of progeny with values greater or less than the values of their parents in segregated generations^60^. Superior lines are referred to as progeny with better values than that of the best parent^127,128^.

### Molecular data for resistance to ascochyta blight

Young leaves were harvested from the F_3:4_ plants and their parents, and then they were stored in a − 20 °C freezer until the date of DNA extraction. Genomic DNA was extracted according to the cetyl-trimethyl bromide (CTAB) protocol^129^. In order to determine the presence of QTLs associated with blight resistance, 152 F_3:4_ lines and their parents were screened by using the markers CaETR-1 and SCY17_590_. While both markers are located on the LG4, CaETR-1 is an ASAP (allele specific associated primers) marker^118^ and linked to QTL_AR1_; SCY17_590_ is a SCAR (sequence characterized amplified region) marker linked to QTL_AR2_^108^. The polymerase chain reaction (PCR) for these analyses contained 1.5 µl of 20 ng/µl DNA, 7.05 µl ddH2O, 1.5 µl 10× buffer, 1.5 µl dNTP’s, 1 µl of 10 pmol primer and 0.2 µl of 5 U/µl Taq DNA polymerase. Amplification was provided in a Blue-Ray Turbo Cycler^®^, which was used throughout this study, programmed for 35 cycles with the following temperature profile: 3 min at 95 °C, 40 s at 50 °C, and 50 s at 72 °C. Cycling was accomplished with a final extension at 72 °C for 5 min. The amplification products were separated on a 3% agarose gel and visualized with ethidium bromide staining.

### Data analyses

All agro-morphological data were analyzed for descriptive statistics using IBM SPSS Statistics^130^ software. F_3_ and F_4_ populations were first divided into two groups according to the leaf shape as imparipinnate (compound, fern-like or normal) and unifoliolate (simple). Then, each group was divided into three groups as single-podded, double-podded and multi-podded according to pods per axil. In this way, six groups were formed. When selecting for the chickpea ideotype, each group was compared with the others. Agro-morphological traits were analyzed using SPSS 22 (IBM SPSS Statistics 2014).

Molecular data were evaluated according to the obtained bands as resistant, susceptible, or the heterozygote by considering the band profiles determined for each primer in previous studies^108,118^.

## Supplementary Information

Supplementary Table S1.

## Data Availability

All data are within the manuscript and supplementary materials.
