# Supplementary material for: A kabuli chickpea ideotype
Source: Sci Rep. 2022 Jan 31;12:1611. doi: 10.1038/s41598-022-05559-3 (PMC8803941; doi:10.1038/s41598-022-05559-3)
Supplement: Supplementary file 1 — Supplementary Table S1. [file 41598_2022_5559_MOESM1_ESM.docx]

**Supplementary Table S1**. Scores of F_3:4_ lines, parental and control genotypes according to two DNA markers (SCY 17_590_ and CaETR-1) linked to ascochyta blight resistance

| **Lines** | **Leaf shape** | **Pods per axil** | **SCY17_590_ (QTL_AR2_)** | **CaETR-1 (QTL_AR1_)** | **100-seed weight (g)** |
| --- | --- | --- | --- | --- | --- |
| 1 | Imparipinnate | double | 1 | 0 | 47.57 |
| 2 | Imparipinnate | double | H | 0 | 45.50 |
| 3 | Unifoliolate | double | 0 | H | 52.50 |
| 4 | Imparipinnate | double | 1 | 0 | 51.50 |
| 5 | Unifoliolate | double | 0 | 0 | 50.00 |
| 6 | Imparipinnate | double | 0 | 0 | 54.00 |
| 7 | Imparipinnate | double | 1 | 0 | 53.40 |
| 8 | Imparipinnate | double | 0 | 0 | 56.70 |
| 9 | Imparipinnate | multi | N/A | 0 | 50.33 |
| 10 | Imparipinnate | double | 0 | 0 | 47.75 |
| 11 | Unifoliolate | multi | H | 0 | 52.30 |
| 12 | Imparipinnate | double | 0 | 0 | 54.00 |
| 13 | Imparipinnate | double | H | 0 | 51.20 |
| 14 | Imparipinnate | double | 0 | 0 | 48.43 |
| 15 | Imparipinnate | double | 0 | 0 | 50.40 |
| 16 | Imparipinnate | double | 1 | 1 | 51.40 |
| 17 | Unifoliolate | double | 1 | 0 | 47.28 |
| 18 | Imparipinnate | double | 1 | 1 | 59.80 |
| 19 | Imparipinnate | double | H | 1 | 45.67 |
| 20 | Imparipinnate | double | 0 | 1 | 48.33 |
| 21 | Imparipinnate | double | 0 | 0 | 46.33 |
| 22 | Unifoliolate | double | 1 | H | 45.71 |
| 23 | Imparipinnate | double | 0 | 0 | 57.00 |
| 24 | Unifoliolate | double | 0 | 1 | 46.83 |
| 25 | Imparipinnate | double | 0 | 1 | 45.67 |
| 26 | Imparipinnate | double | 0 | 0 | 53.80 |
| 27 | Imparipinnate | double | 0 | H | 45.90 |
| 28 | Imparipinnate | double | 0 | H | 45.87 |
| 29 | Imparipinnate | multi | 0 | 0 | 52.57 |
| 30 | Imparipinnate | double | 0 | 1 | 47.67 |
| 31 | Imparipinnate | double | 0 | 1 | 46.33 |
| 32 | Imparipinnate | double | 0 | 0 | 52.50 |
| 33 | Imparipinnate | double | 0 | 0 | 50.00 |
| 34 | Imparipinnate | double | 0 | N/A | 56.33 |
| 35 | Unifoliolate | double | 0 | H | 46.14 |
| 36 | Unifoliolate | double | 1 | 0 | 47.00 |
| 37 | Imparipinnate | double | 0 | H | 46.25 |
| 38 | Imparipinnate | double | H | 0 | 49.75 |
| 39 | Imparipinnate | multi | 0 | 0 | 59.30 |
| 40 | Imparipinnate | double | 1 | 1 | 57.50 |
| 41 | Imparipinnate | double | 0 | H | 45.57 |
| 42 | Imparipinnate | multi | H | H | 46.85 |
| 43 | Imparipinnate | double | 1 | 0 | 45.57 |
| 44 | Imparipinnate | multi | 1 | 0 | 45.57 |
| 45 | Imparipinnate | double | 0 | 0 | 47.60 |
| 46 | Imparipinnate | double | 0 | H | 51.14 |
| 47 | Unifoliolate | double | 1 | 0 | 47.72 |
| 48 | Imparipinnate | double | 0 | 0 | 48.86 |
| 49 | Imparipinnate | double | 0 | 0 | 50.40 |
| 50 | Unifoliolate | double | 1 | 0 | 49.75 |
| 51 | Imparipinnate | double | 1 | H | 56.00 |
| 52 | Imparipinnate | double | 1 | 0 | 47.17 |
| 53 | Imparipinnate | double | 0 | 0 | 46.67 |
| 54 | Unifoliolate | double | 1 | 0 | 50.25 |
| 55 | Imparipinnate | double | 0 | 1 | 49.80 |
| 56 | Imparipinnate | double | 0 | 1 | 55.67 |
| 57 | Imparipinnate | double | 1 | 1 | 46.70 |
| 58 | Imparipinnate | double | 0 | 0 | 52.00 |
| 59 | Imparipinnate | double | 1 | 0 | 53.25 |
| 60 | Imparipinnate | double | 0 | H | 48.40 |
| 61 | Unifoliolate | double | 0 | 1 | 47.85 |
| 62 | Imparipinnate | double | 0 | H | 54.00 |
| 63 | Unifoliolate | double | 0 | H | 51.20 |
| 64 | Unifoliolate | double | 0 | 0 | 47.40 |
| 65 | Imparipinnate | double | 1 | 0 | 62.50 |
| 66 | Unifoliolate | double | 1 | 0 | 45.50 |
| 67 | Imparipinnate | double | 1 | 0 | 56.50 |
| 68 | Imparipinnate | double | 1 | 0 | 47.50 |
| 69 | Imparipinnate | double | 0 | 0 | 53.00 |
| 70 | Imparipinnate | double | 1 | 0 | 49.90 |
| 71 | Imparipinnate | double | 0 | 0 | 54.67 |
| 72 | Imparipinnate | double | 0 | 0 | 51.00 |
| 73 | Imparipinnate | double | 0 | 1 | 46.30 |
| 74 | Unifoliolate | double | 1 | 0 | 45.00 |
| 75 | Imparipinnate | double | H | 1 | 48.80 |
| 76 | Imparipinnate | double | 1 | 0 | 52.40 |
| 77 | Unifoliolate | double | 0 | 0 | 53.30 |
| 78 | Imparipinnate | double | H | 0 | 45.20 |
| 79 | Unifoliolate | multi | 0 | 0 | 53.75 |
| 80 | Imparipinnate | double | 0 | 0 | 49.83 |
| 81 | Imparipinnate | double | 1 | 0 | 50.20 |
| 82 | Unifoliolate | double | 0 | 0 | 49.60 |
| 83 | Imparipinnate | double | 1 | 0 | 51.60 |
| 84 | Imparipinnate | double | 0 | 0 | 45.72 |
| 85 | Imparipinnate | double | 1 | 0 | 47.71 |
| 86 | Imparipinnate | double | 0 | 0 | 45.80 |
| 87 | Imparipinnate | double | 0 | 0 | 46.50 |
| 88 | Imparipinnate | double | 0 | 0 | 45.80 |
| 89 | Imparipinnate | multi | 0 | N/A | 55.00 |
| 93 | Imparipinnate | double | 0 | H | 55.00 |
| 90 | Imparipinnate | double | 0 | H | 46.12 |
| 94 | Imparipinnate | double | 1 | 0 | 46.66 |
| 91 | Unifoliolate | double | 1 | 0 | 50.60 |
| 95 | Imparipinnate | double | 0 | 0 | 46.80 |
| 92 | Imparipinnate | double | 0 | 0 | 61.33 |
| 96 | Unifoliolate | double | H | 0 | 46.75 |
| 97 | Imparipinnate | double | 1 | 0 | 46.40 |
| 98 | Unifoliolate | double | 1 | 0 | 49.33 |
| 99 | Imparipinnate | double | 1 | 0 | 52.33 |
| 100 | Imparipinnate | double | 1 | 0 | 45.66 |
| 101 | Imparipinnate | double | 0 | 0 | 50.40 |
| 102 | Imparipinnate | double | 1 | 0 | 47.72 |
| 103 | Unifoliolate | double | 0 | 0 | 45.25 |
| 104 | Unifoliolate | double | 0 | 0 | 57.75 |
| 105 | Imparipinnate | double | 0 | 0 | 49.75 |
| 106 | Unifoliolate | multi | 1 | 1 | 46.20 |
| 107 | Unifoliolate | double | 0 | 0 | 57.66 |
| 108 | Imparipinnate | double | 1 | 0 | 50.20 |
| 109 | Unifoliolate | double | 0 | 0 | 47.00 |
| 110 | Unifoliolate | double | 0 | 1 | 45.00 |
| 111 | Unifoliolate | double | 0 | 0 | 55.00 |
| 112 | Unifoliolate | double | 1 | 0 | 49.20 |
| 113 | Imparipinnate | double | 1 | 0 | 50.25 |
| 114 | Imparipinnate | double | 1 | 0 | 53.25 |
| 115 | Imparipinnate | double | 1 | 1 | 56.60 |
| 116 | Imparipinnate | double | 1 | 0 | 47.00 |
| 117 | Unifoliolate | double | 1 | 0 | 47.50 |
| 118 | Imparipinnate | double | 0 | 0 | 46.00 |
| 119 | Unifoliolate | multi | 1 | 0 | 49.70 |
| 120 | Imparipinnate | double | 0 | 0 | 58.50 |
| 121 | Unifoliolate | double | 1 | 0 | 49.75 |
| 122 | Unifoliolate | double | 1 | 1 | 46.80 |
| 123 | Unifoliolate | double | 1 | 0 | 50.25 |
| 124 | Imparipinnate | double | 0 | 1 | 49.87 |
| 125 | Imparipinnate | double | 0 | 1 | 50.60 |
| 126 | Imparipinnate | double | 0 | 0 | 48.20 |
| 127 | Imparipinnate | double | 0 | 0 | 50.60 |
| 128 | Imparipinnate | double | 0 | 0 | 47.60 |
| 129 | Unifoliolate | double | 1 | 1 | 46.80 |
| 130 | Unifoliolate | double | N/A | N/A | 50.16 |
| 131 | Imparipinnate | double | 0 | 0 | 49.50 |
| 132 | Imparipinnate | double | 1 | 1 | 50.20 |
| 133 | Imparipinnate | double | 1 | 0 | 52.75 |
| 134 | Imparipinnate | double | 0 | 0 | 48.16 |
| 135 | Imparipinnate | double | 0 | 0 | 53.00 |
| 136 | Imparipinnate | double | 0 | 0 | 45.50 |
| 137 | Imparipinnate | double | 0 | 1 | 53.00 |
| 138 | Imparipinnate | double | 0 | 0 | 51.60 |
| 139 | Imparipinnate | double | 1 | 0 | 49.60 |
| 140 | Imparipinnate | double | 0 | 0 | 45.50 |
| 141 | Imparipinnate | double | 0 | 1 | 45.00 |
| 142 | Imparipinnate | double | 0 | 0 | 45.83 |
| 143 | Unifoliolate | double | 0 | 0 | 45.80 |
| 144 | Imparipinnate | double | 0 | 0 | 51.00 |
| 145 | Imparipinnate | double | 0 | 0 | 53.60 |
| 146 | Unifoliolate | double | 1 | 0 | 49.75 |
| 147 | Imparipinnate | double | H | 0 | 50.20 |
| 148 | Imparipinnate | double | 0 | 1 | 47.13 |
| 149 | Unifoliolate | double | 0 | 1 | 49.75 |
| 150 | Unifoliolate | double | 0 | 0 | 45.20 |
| 151 | Unifoliolate | double | H | 0 | 46.20 |
| 152 | Imparipinnate | double | 1 | 0 | 48.00 |
| Sierra | Unifoliolate | single | 0 | 1 | 46.90 |
| CA 2969 | Imparipinnate | double | 1 | 0 | 27.00 |
| ILC 1929 | Imparipinnate | single | 0 | 0 | 40.90 |
| ILC 3279 | Imparipinnate | single | 1 | 1 | 27.50 |
